# Daily cytokine fluctuations, driven by leptin, are associated with fatigue severity in chronic fatigue syndrome: evidence of inflammatory pathology

**DOI:** 10.1186/1479-5876-11-93

**Published:** 2013-04-09

**Authors:** Elizabeth Ann Stringer, Katharine Susanne Baker, Ian R Carroll, Jose G Montoya, Lily Chu, Holden T Maecker, Jarred W Younger

**Affiliations:** 1 Department of Anesthesiology, Stanford University School of Medicine, Stanford, CA, 94304, USA; 2 Department of Medicine, Infectious Diseases, Stanford University School of Medicine, Stanford, CA, 94304, USA; 3 Department of Microbiology & Immunology, Stanford University School of Medicine, Stanford, CA, 94304, USA; 4 1070 Arastradero Road, Suite 200, Palo Alto, CA, 94304-1336, USA; 5 Independent Consultant, Stanford, CA, 94304, USA

**Keywords:** Chronic fatigue syndrome, Cytokines, Leptin, Daily immune monitoring

## Abstract

**Background:**

Chronic fatigue syndrome (CFS) is a debilitating disorder characterized by persistent fatigue that is not alleviated by rest. The lack of a clearly identified underlying mechanism has hindered the development of effective treatments. Studies have demonstrated elevated levels of inflammatory factors in patients with CFS, but findings are contradictory across studies and no biomarkers have been consistently supported. Single time-point approaches potentially overlook important features of CFS, such as fluctuations in fatigue severity. We have observed that individuals with CFS demonstrate significant day-to-day variability in their fatigue severity.

**Methods:**

Therefore, to complement previous studies, we implemented a novel longitudinal study design to investigate the role of cytokines in CFS pathophysiology. Ten women meeting the Fukuda diagnostic criteria for CFS and ten healthy age- and body mass index (BMI)-matched women underwent 25 consecutive days of blood draws and self-reporting of symptom severity. A 51-plex cytokine panel via Luminex was performed for each of the 500 serum samples collected. Our primary hypothesis was that daily fatigue severity would be significantly correlated with the inflammatory adipokine leptin, in the women with CFS and not in the healthy control women. As a post-hoc analysis, a machine learning algorithm using all 51 cytokines was implemented to determine whether immune factors could distinguish high from low fatigue days.

**Results:**

Self-reported fatigue severity was significantly correlated with leptin levels in six of the participants with CFS and one healthy control, supporting our primary hypothesis. The machine learning algorithm distinguished high from low fatigue days in the CFS group with 78.3% accuracy.

**Conclusions:**

Our results support the role of cytokines in the pathophysiology of CFS.

## Background

Chronic fatigue syndrome (CFS) is a debilitating disorder characterized by at least six months of persistent fatigue that is not alleviated by rest [1]. Individuals with CFS often report additional symptoms such as muscle pain, joint pain, headaches, poor sleep, inability to concentrate, sore throat, swollen lymph nodes, and post-exertion malaise [2]. The condition affects an estimated 42 out of every 10,000 – equating to approximately one million – individuals in the United States [3]. In addition to the impact on quality of life, it presents substantial costs to the economy in both direct medical expenditures and lost work productivity [3]. The majority of diagnosed individuals are adult women [4].

The development of effective treatments for CFS has been hindered by the lack of a clearly identified pathophysiological mechanism for the disorder. There are no objective blood tests to confirm a diagnosis, and no well-accepted targets for intervention. Several studies, however, have demonstrated abnormal inflammatory processes in CFS. Cross-sectional analyses (CFS versus healthy control) have identified elevated levels of proinflammatory cytokines such as TNF-alpha, IL-1alpha, IL-1beta, and IL-6 [5,6]. Results, however, are inconsistent across studies [7], and no potential biomarkers have been consistently supported by research to advance diagnosis or treatment.

One challenge of research to date is that individuals with CFS exhibit significant day-to-day variability in their fatigue severity. This within-subject variability may reduce the sensitivity of cross-sectional studies to consistently identify immune abnormalities in these individuals [8]. We have therefore tested a within-person, daily immune monitoring approach for identifying CFS biomarkers. By measuring immune markers over many consecutive days, we sought to identify serum analytes that “track” increases and decreases in fatigue severity. This intensive longitudinal approach may reveal novel biomarkers overlooked by traditional cross-sectional immune studies and unveil novel mechanisms in CFS pathogenesis. The technique may be particularly useful in cases where daily symptom variability introduces statistical noise in cross-sectional data, or when immune factors exist in normal concentrations, but still drive pathological processes because of sensitized targets. Our goal with this approach is to identify biomarkers that facilitate diagnosis and may be targets of future therapies.

We previously piloted the daily immune monitoring approach in three women with fibromyalgia (FM) and co-morbid CFS. The participants were monitored for 25 consecutive days, and their daily serum samples analyzed for concentrations of 51 different cytokines. Out of the 51 analytes, only leptin was significantly correlated with day-to-day self-reported fatigue severity in all three women [9]. Leptin is an analyte of interest because, in addition to being an appetite-regulatory hormone, the adipokine is an inflammatory agent that has been linked to pathological inflammatory fatigue [10,11]. Leptin provokes the release of proinflammatory cytokines from many cell types, including centrally acting microglia [12], and is a mediator of cytokine-induced sickness behavior [13].

Given the results of our preliminary data, we designed a study comparing ten women with CFS to ten healthy, age-, sex- and BMI-matched controls. Participants underwent 25 consecutive days of blood draws, and completed reports of fatigue severity twice a day. We hypothesized that leptin would be associated with daily fatigue severity in the participants with CFS but not in healthy controls. As a secondary, exploratory aim, we also tested the ability of 50 other immune factors to predict fatigue variability in both groups. To our knowledge, this is the first study of leptin and its role in CFS.

## Methods

### Participant selection

Healthy women and women with CFS, meeting the Fukuda diagnostic criteria case definition [1], were recruited from an existing, prescreened database maintained by Stanford’s CFS Research Team. During an initial phone screen we identified potential participants between the ages of 18 and 62 who were fluent in English, capable of transporting themselves to Stanford daily, and able to receive daily venous blood draws, for 25 days in a row. Exclusionary criteria included untreated or uncontrolled significant psychological comorbidities, blood or clotting disorders, rheumatologic disease, autoimmune disease, Lyme’s disease, elevated viral load, active infection, smoking, pregnancy or plans to become pregnant, and the use of blood thinning medications or antibiotics. Because inflammation increases with age, and circulating leptin increases with BMI, healthy control participants were age-matched and BMI-matched to the participants with CFS. During the phone screen, 33 patients were excluded from participation due to conflicts with daily transportation to Stanford (16), Lyme’s disease (6), Hashimoto’s disease (3), not meeting diagnostic criteria (3), resolved symptoms (2), sex (2), and age (1). Twelve healthy controls were excluded for pain conditions (4), thyroid conditions (3), conflicts with daily transportation to Stanford (2), age- and BMI-matching (2), and smoking (1).

Participants were screened again during a site visit at Stanford’s Adult and Pediatric Pain Laboratory. Each participant completed screening questionnaires and blood tests. The questionnaires included a demographic form, the Hospital Anxiety and Depression Scale (HADS) [14], and the Fibromyalgia Assessment Form [15]. A score of 16 or higher on the HADS Depression subscale was exclusionary. Participants were also excluded if the blood tests showed erythrocyte sedimentation rate (ESR) greater than 60 mm/hr, thyroid stimulating hormone (TSH) outside the range of 0.4 – 4 mIU/L, C-reactive protein (CRP) over 0.9 mg/dL, positive rheumatoid factor, or positive anti-nuclear antibody (ANA).

Twelve patients and eleven controls met the study inclusion/exclusion criteria, and each provided written informed consent for this study in accordance with a protocol approved by the Stanford University Institutional Review Board. One patient withdrew participation for reasons unrelated to the study, and one for privacy concerns. One control withdrew participation due to an unrelated accident affecting her ability to drive to Stanford for daily blood draws. Results for the twenty completers are reported. The study was run between September 2011 and October 2012.

### Study design

The study protocol consisted of a two-week observational baseline period followed by 25 consecutive days of blood draws. At the site screening visit, participants were given an Android-based device equipped with software (Dooblo’s SurveyToGo, Kefar Sava, Israel) to assess the severity of their CFS symptoms on a visual analogue scale (VAS). The primary outcome, daily fatigue severity, was assessed by asking, “Overall, how severe has your fatigue been today?” The far left of the scale was anchored at “no fatigue” and the far right was anchored at “severe fatigue”. Similar questions about the severity of muscle and joint pain, as well as the quality of sleep were included in the assessment. Participants completed the measures twice per day – once in the morning and once at night. Following the two-week observational phase, participants underwent 25 consecutive daily visits to Stanford’s Clinical and Translational Research Unit (CTRU) for their blood draws, and continued completing the symptom severity surveys twice a day throughout this period. During the CTRU visits, participants’ vital signs (blood pressure, heart rate, and body temperature) were also taken to assess general health and to screen for acute infection.

Blood was drawn by trained phlebotomists or research nurses with a 23-gauge butterfly needle into two 4 cc clot-activator tubes. The site of the blood draw was rotated daily to minimize participant discomfort and maintain vein integrity. After 30 minutes at room temperature, the blood samples were centrifuged at 350 × g for 15 minutes, and the serum was extracted, divided into four cryovials, and stored in a -80 °C freezer for later testing. For each participant, CTRU visits were held within a two-hour window (or narrower) throughout the protocol to control for known diurnal fluctuations in cytokines [16,17]. Individual schedule conflicts prevented routine appointments in only a few cases. If unforeseen events prevented participants from keeping an appointment (one patient and two healthy controls missed between one and three appointments each), a blood draw for each missed appointment was added to the end of the 25 day protocol so that a total of 25 blood samples were collected for each of the twenty participants.

### Sample processing

Serum samples were processed by Stanford’s Human Immune Monitoring Center. Human 51-plex Luminex kits were purchased from Affymetrix and used according to the manufacturer’s recommendations, with modifications as described below. Briefly, samples were mixed with antibody-linked polystyrene beads on 96-well filter-bottom plates and incubated at room temperature for 2 hours, followed by overnight incubation at 4°C. Plates were vacuum filtered and washed twice with wash buffer, then incubated with biotinylated detection antibody for 2 hours at room temperature. Samples were then filtered and washed twice as above and resuspended in streptavidin-PE. After incubation for 40 minutes at room temperature, two additional vacuum washes were performed, and the samples resuspended in Reading Buffer. Each sample was measured in singlet. Plates were read using a Luminex 200 instrument with a lower bound of 100 beads per sample per cytokine. Median fluorescence intensity (MFI) values were reported for each cytokine, using Masterplex software (Hitashi Corp.), after data quality control to remove samples with low bead counts or other technical abnormalities.

### Statistical analysis

All data were processed and analyzed in SPSS Statistics 20 (Armonk, NY: IBM Corp) unless otherwise noted. Values of cytokine MFI and fatigue severity were first subject-centered using z-score transformation. The z-scores served two purposes. First, they allowed cytokine levels and fatigue severity to be plotted on the same scale in order to visualize trends. Second, they allowed analyses to be run between subjects, without being adversely affected by large baseline between-subject variability.

Daily data (cytokines and fatigue) were temporally smoothed with a 3-day moving average. The moving average is a procedure commonly employed with time series data, to remove high-frequency variability and minimize the impact of random noise on detecting significant trends [18]. Temporally smoothed data were used in all analyses except for cytokine network mapping.

NodeXL [19] was used to construct and depict a network diagram of fatigue and 51 cytokines, creating a visual display of the relationships among variables for both the CFS and control groups. Group bivariate correlations were fed into a spring-embedded Fruchterman-Reingo algorithm in NodeXL. Variables (fatigue and cytokines) are represented by squares or “nodes,” and variables that are significantly correlated are connected by lines or “vectors.” The *p*-value was adjusted for multiple comparisons using a 0.01 false-discovery rate, which yielded a corrected *p* < 0.0012 statistical threshold for each of the links displayed in the network diagram. For visualization in both diagrams, fatigue and leptin are highlighted in red. In the CFS diagram, the relationships between leptin and other cytokines that are statistically significant are also highlighted by red vertices. The multitude of vertices within the network diagrams illustrates the degree to which inflammatory factors fluctuate together.

In a separate analysis, as a post-hoc, proof-of-concept test, we utilized a machine learning algorithm in Weka [20] to test the ability of cytokines to distinguish high from low fatigue days. Our goal was to determine whether cytokines alone could accurately predict daily fatigue severity in participants with CFS. Machine learning algorithms use multivariate approaches to achieve greater sensitivity than massive univariate tests for identifying complex predictor-outcome relationships. For each of the ten participants with CFS, the dataset included the nine most severe fatigue days and nine least severe fatigue days, for a total of 180 cases. We used Weka’s LibLINEAR support vector machine algorithm with a cost function C = 1 and a 10-fold independent cross-validation.

The model built for the participants with CFS was also tested on the control group to see if this trained model could also predict fatigue in healthy individuals. For the healthy controls, the data were likewise divided into the nine most severe and nine least severe days, but fatigue scores that were the same for both high and low fatigue days were excluded. For example, two participants rated their fatigue as “0” for each of the 25 days, so their data were excluded from this analysis. A total of 136 cases were used for the controls.

## Results

### Demographics

Twenty women between the ages of 29 and 62 (CFS mean: 52.9; control mean: 53.0) were included in the primary analyses. Table 1 presents the basic recommended [21] demographic and medical inventory for each participant with CFS (#1 – 10) and for each of the healthy controls (#11 – 20). All of the participants classified themselves as Caucasian except for three of the healthy controls who self-identified as Asian, African- American, and Latina. Two CFS participants were employed at the time of participation, whereas eight of the healthy controls were employed. During the two-week baseline phase, the mean VAS values for fatigue, muscle pain, and joint pain were significantly higher in participants with CFS than in controls, while the controls had higher sleep quality scores (Table 1).

**Table 1:** Data elements

**Participant**	**Age (years)**	**Race/ Ethnicity**	**Income range**	**Employment/Disability status**	**BMI**	**Mode of onset**	**Duration of illness (years)**	**Fatigue severity (0=none, 100=severe)**	**Muscle pain severity (0=none, 100=severe)**	**Joint pain severity (0=none, 100=severe)**	**Sleep quality (0=poor, 100=good)**	**Multi-dimensional fatigue inventory MFI-20**^**§**^	**Fatigue severity scale**^**§**^	**HADS: Depression Sub-score**	**Meets FM criteria**	**Co-morbid conditions**	**Medications**
1	54	Caucasian	$10,000-19,999	Not reported	18	Acute	35	61	50	46	39	88	6.67	6	Yes	Osteoarthritis	Aspirin, Levothyroxine
2	53	Caucasian	$20,000-29,999	Retired/Disablility	31.6	Acute	15	73	71	70	52	96	6.78	13	Yes	Hypothyroid, High Blood Pressure	Alprazolam, Amlodipine, Levothyroxine, Valganciclovir^†^, Naproxen, Melatonin, Aspirin, Estradiol
3	53	Caucasian	Not reported	Retired	25.2	Gradual	30	80	60	41	65	76	6.56	6	Yes	Degenerate Arthritis, Spinal Stenosis, Sleep Apnea	Estradiol, Valganciclovir^†^, Lidocaine
4	47	Caucasian	less than $10,000	Unemployed	25.9	Acute	9	31	6	50	41	81	6.67	3	Yes	High Cycling EBV and HHV-6, Chronic Sinus Infections, Chlamidophila Pneumonia	Loratadine, Cetirizine, Hydroxyzine, Diphenhydram, Gabapentin, 5- Hydroxytryptophan, St. John's Wort
5	61	Caucasian	$10,000-19,999	Unemployed/Disability	35.7	Gradual	7	50	33	32	37	94	7.00	10	No	Graves Disease/Hypothyroid, Obesity	Bupropion, Estradiol, Fenofibrate, Levothyroxine, Valganciclovir†, Fluoxetine, Acetaminophen
6	60	Caucasian	$80,000 or more	Employed	20.6	Gradual	5	64	49	42	39	90	5.89	11	Yes	None	None
7	51	Caucasian	$10,000-19,999	Disability	23.3	Gradual	5	81	25	0	71	89	5.89	3	Yes	None	Acyclovir^††^
8	29	Caucasian	$40,000-49,999	Employed	27.5	Gradual	15	62	63	67	55	NA	NA	0	Yes	None	Topiramate
9	55	Caucasian	$60,000-69,999	Retired/Disablility	20.4	Acute	10	74	70	48	55	NA	NA	14	Yes	None	Bupropion, Controlled-Release Morphine, Progesterone, Loratadine, Diazepam, Alprazolam, Estradiol, Sertraline, Zolpidem, Rizatriptan, Ibuprofen, Ketorolac
10	62	Caucasian	$30,000-39,99	Not reported	42	Gradual	20	36	25	8	44	84	6.56	3	No	Hypothyroid, Obesity, High Blood Pressure, ADHD	Olmesartan, Bupropion, Thyroid, Estradiol, Dextroamphetamine and Amphetamine
11	57	Caucasian	$20,000-29,999	Employed	33.1	NA	NA	0	0	0	71	42	2.22	0	No	High Blood Pressure	Amlodipine, Asprin
12	48	Caucasian	less than $10,000	Unemployed	23	NA	NA	18	23	4	70	NA	NA	1	No	None	None
13	51	Caucasian	less than $10,000	Employed	21.3	NA	NA	30	7	19	54	NA	NA	2	No	None	Prozac
14	58	Caucasian	less than $10,000	Employed	25.1	NA	NA	0	0	0	67	NA	NA	3	No	None	None
15	52	Asian	Not reported	Employed	19.7	NA	NA	6	1	0	64	48	1.88	0	No	None	Proton Pump Inhibitor
16	50	Caucasian	$50,000-59,999	Employed	20.5	NA	NA	0	0	0	96	20	1.22	0	No	None	None
17	44	Caucasian	$30,000-39,999	Employed	25.5	NA	NA	5	12	0	77	25	1.77	1	No	None	None
18	47	African American	$80,000 or more	Employed	31.6	NA	NA	19	9	1	80	28	1.66	2	No	Borderline High Blood Pressure, High Cholesterol	Zyrtec
19	61	Caucasian	$40,000-49,999	Retired	24	NA	NA	15	0	0	79	26	1.77	1	No	Plantar Fasciitis	Loratadine
20	62	Latina	$10,000-19,999	Employed	24.5	NA	NA	6	5	0	79	22	1.33	1	No	None	None

Data elements for each of the 20 participants (CFS: #1 – 10, Controls: #11 – 20). Participants self-reported race/ethnicity, income, employment/disability status, height, weight, mode of CFS onset, duration of CFS, co-morbid conditions, and medications. Fatigue, muscle pain, joint pain, and sleep quality were determined by taking the mean from the two-week baseline period of symptom severity assessments. The HADS [14] and Fibromyalgia Assessment Form [15] were administered during the screening visit. ^§^Scores are from an existing database maintained by Stanford’s CFS Research Team. ^†^Valganciclovir was prescribed as an experimental treatment for CFS, ^††^Acyclovir was prescribed for HSV-1 and HSV-2. NA = Not Available.

Four participants with CFS reported an acute onset and six reported a gradual onset of illness, and the average duration of illness was 15 years. Eight women with CFS also met the criteria for a diagnosis of Fibromyalgia, based on the recent self-report American College of Rheumatology criteria [15]. None of the women met criteria for severe depression based on the HADS Depression sub-scale, and any co-morbid psychological conditions were stable and maintained by medication. Table 1 also includes other co-morbid conditions and medications for all participants. Individuals with higher BMI tended to have higher serum leptin concentrations, CFS: *r* = 0.614, *p* = 0.059; controls: *r* = 0.863, *p* = 0.001. There was no significant difference between groups for BMI (*t* = -0.784, *p* = 0.443) or leptin (*t* = -0.867, *p* = 0.397).

## Main results

**Figure 1:**
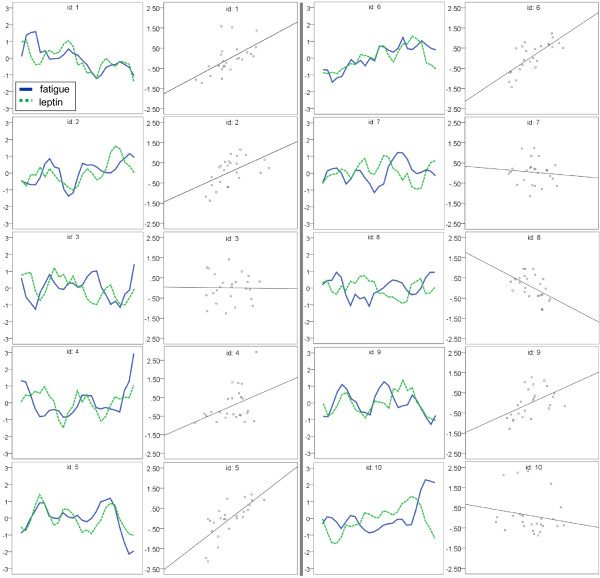
**Fatigue and leptin plots for participants with CFS.** Left panes: Self-reported fatigue severity (solid blue line) "tracks" with serum leptin concentration (dotted green line) over the 25-day protocol in six patients with CFS (#'s 1, 2, 4, 5, 6, 9). Right panes: Scatter plots illustrate the strength of association between self-reported fatigue severity and serum leptin concentration. The data have been Z-score transformed to fit on the same scale. The mean AM and PM fatigue scores are displayed. Note for #4, the y-axis scale extends to 2.95 instead of 2.5 to accommodate a data point.

**Figure 2:**
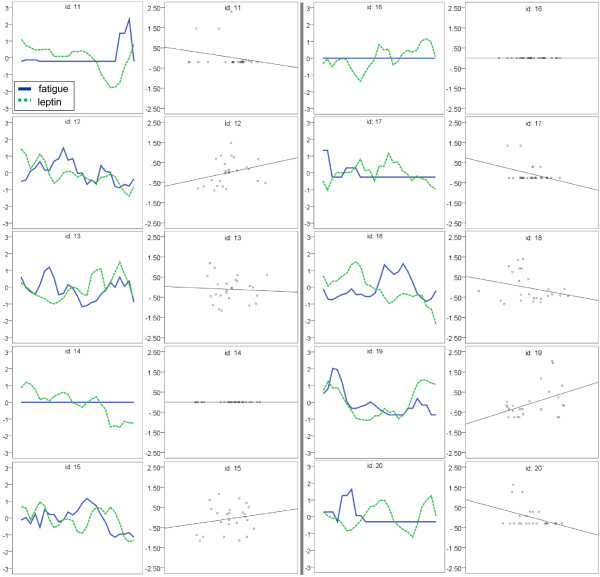
**Fatigue and leptin plots for healthy controls.** Left panes: Self-reported fatigue severity (solid blue line) "tracks" with serum leptin concentration (dotted green line) over the 25-day protocol in one healthy control (#19). Right panes: Scatter plots illustrate the strength of association between self-reported fatigue severity and serum leptin concentration. The data have been Z-score transformed to fit on the same scale. The mean AM and PM fatigue scores are displayed.

Our primary hypothesis was that leptin would predict daily fatigue severity in patients with CFS but not in healthy controls. Daily fluctuations in self-reported fatigue (N = 250) were significantly correlated with daily leptin levels in participants with CFS, *r* = 0.303, *p* < 0.01. In contrast, healthy control data (N = 250) showed no correlation between fatigue and leptin, *r* = -0.010, *p* < 0.873. Figure 1 displays the 25-day sequence charts of all ten participants with CFS, as well as corresponding scatterplots showing the degree of association between leptin and fatigue, and Figure 2 for the ten healthy controls. A significant positive correlation was observed between leptin and fatigue in six participants with CFS (Table 2: #’s 1, 2, 4, 5, 6, 9), with *r*’s ranging between 0.407 and 0.747, and one healthy control (Table 2: #19). A significant negative correlation was observed in one individual with CFS (Table 2: #8). Leptin correlations with fatigue severity scores collected at different times of day (AM, PM, and mean) for each participant are displayed in Table 2.

**Table 2:** Outcome measures

	Pearson correlation coefficient with Leptin (*p<0.05; **p<0.01)		
**Participant**	**AM fatigue**	**PM fatigue**	**Mean fatigue**
1	0.38	0.696**	0.582**
2	0.32	0.676**	0.535**
3	0.02	−0.047	−0.015
4	0.304	0.451*	0.407*
5	0.588**	0.749**	0.731**
6	0.583**	0.434*	0.747**
7	−0.173	0.005	−0.097
8	−0.405	* 0.034	−0.516**
9	0.591**	0.250	0.497*
10	−0.015	−0.205	−0.173
11	0.298	−0.237	−0.226
12	0.095	0.238	0.276
13	−0.416*	0.545*	−0.056
14	NA	NA	NA
15	0.069	0.222	0.176
16	NA	NA	NA
17	−0.402*	−0.241*	−0.342
18	−0.210	−0.327	−0.268
19	0.262	0.539**	0.429*
20	−0.363	−0.412*	−0.386

Outcome measures for each of the 20 participants (CFS: #1 – 10, Controls: #11 – 20). The correlations (*r*-value) between serum leptin levels and daily (AM, PM, and mean) fatigue severity are reported, *p<0.05, **p<0.01.

**Figure 3:**
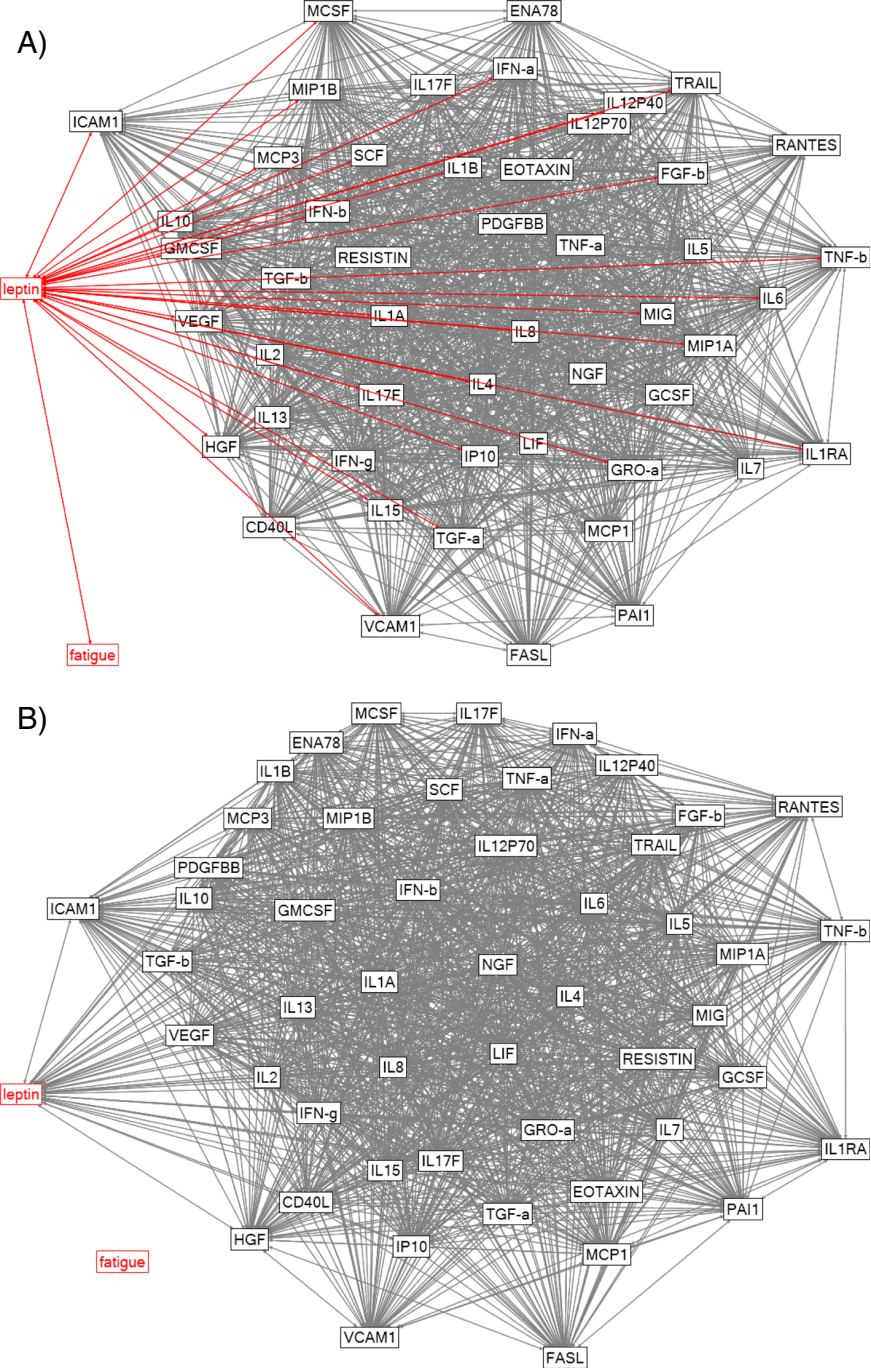
**Network diagrams for A) participants with CFS and B) healthy controls.** A network diagram of fatigue and 51 cytokines was constructed in NodeXL. All participants were included in the analysis. Fatigue and leptin are highlighted in red, and variables with significant correlations with fatigue and leptin are represented by red edges (**A**). The network diagram was thresholded at *p* < 0.0012, false discovery rate controlled for multiple comparisons.

Next, in the CFS group and control group, we examined relationships between fatigue and all 51 available cytokines. These analyses were intended to determine whether analytes other than leptin are important in predicting daily fluctuations in fatigue. First, bivariate correlations were computed between all variables and graphed for the CFS group (Figure 3A) and the control group (Figure 3B). Of the 51 analytes, only leptin was directly correlated with fatigue in the CFS group, using the *p*-value threshold corrected for multiple comparisons. Leptin, in turn, was significantly associated with 29 other cytokines, with the strongest relationships including granulocyte macrophage colony-stimulating factor (GMCSF), macrophage colony-stimulating factor (MCSF), transforming growth factor alpha (TGF-a), interferon-alpha (IFN-a), interferon-beta (IFN-b), intercellular adhesion molecule 1 (ICAM1), monocyte-specific chemokine 3 (MCP3), interleukin 6 (IL6), interleukin 10 (IL10), interleukin 12 subunit at p40 (IL12 P40), tumor necrosis factor beta (TNF-b), TNF-related apoptosis-inducing ligand (TRAIL), and vascular endothelial growth factor (VEGF). These analytes were associated with a number of other analytes, leading to a “nest” of related cytokines. These tertiary relationships support the overall role of inflammation in the pathophysiology of fatigue in patients with CFS. In contrast, no analytes correlated with fatigue in the control group.

In order to determine how well we could classify days as high fatigue or low fatigue using only cytokines as predictors, we used a machine learning approach to build a predictive model. In the independent validation for the CFS group, Weka’s LibLINEAR algorithm distinguished high fatigue from low fatigue days (N = 180) with 78.3% accuracy. The CFS model correctly identified 77.8% of the low fatigue days and 78.9% of high fatigue days.

We next applied the CFS-trained model to the control dataset (N = 136), and the model performed at chance with 55.15% accuracy. The model correctly identified 43.1% of low fatigue days and 68.8% of high fatigue days in the healthy controls. This suggests that different factors drive fatigue severity in patients with CFS.

## Discussion

We demonstrated that daily fatigue severity is significantly correlated with daily serum leptin levels in women with CFS but not in healthy controls. Six participants with CFS and one healthy control demonstrated significant positive correlations between fatigue and leptin. The findings confirmed our primary hypothesis that leptin predicts daily fatigue levels in women with CFS. In a secondary analysis, we widened the analyses to 50 other cytokines. In participants with CFS the relationship between immune factors and fatigue was so strong that we were able to distinguish low fatigue days from high fatigue days with 78.3% accuracy, using only cytokines as predictors. Additionally, when this CFS-trained model was applied to the control dataset, the model performed at a chance level. The results support a role of inflammation in CFS pathology.

In the search to uncover CFS pathophysiological mechanisms, considerable effort has been directed at examining dysregulation of the immune system. Of particular interest are cytokines that can drive sickness behaviors such as fatigue and hypersensitivity to pain [22,23]. While many studies have identified potential cytokine differences between individuals with CFS and healthy controls, results have been contradictory [7]. The lack of consistent results across studies may be due partially to the use of cross-sectional designs in a condition driven by atypical, low-level inflammatory processes [8]. We have observed that CFS symptom severity can change drastically in a short period of time, with abrupt shifts over just a couple of days. Such daily variability may blur the distinction between cases and controls in cross-sectional studies. We therefore propose a novel approach of daily immune monitoring to complement conventional cross-sectional studies. By capturing immune fluctuations every day, we can detect immune-fatigue relationships even when cytokine concentrations are highly variable over time, or when cytokines are driving symptoms at “normal” concentrations because downstream targets have been sensitized.

In our analyses of participants with CFS, the strongest relationship between cytokines and fatigue involved leptin. Although leptin is most broadly recognized as a pleiotropic peptide hormone secreted by adipocytes for regulating energy homeostasis, it is also an adipokine that modulates immune responses. Administration of endotoxin in both rodents and humans leads to increased gene expression for leptin and increased serum leptin levels [24,25]. Likewise, leptin administration affects both the adaptive and innate immune systems [26], increasing the release of the proinflammatory cytokines TNF-alpha, IL-2, IL-6, and IL-12 [27,28]. Multiple studies have demonstrated elevated levels of circulating leptin in chronic inflammatory conditions [29,30]. Serum leptin concentrations are also associated with fatigue severity in patients with chronic hepatitis C and irritable bowel syndrome [10,11].

The relationship we observed between leptin and fatigue existed even though leptin levels were not abnormally elevated, and there was no statistical difference in leptin values between the CFS and control groups. Most of the body’s leptin is secreted from white adipose tissue, and consequently, circulating leptin levels should be correlated with BMI. Leptin also demonstrates regular diurnal fluctuations that can change concentrations by over 50% in the course of a day [16,17]. To mitigate the effect of diurnal rhythms on our results, we asked that participants complete their laboratory session at the same time each day, within a two-hour window. The percent change of leptin we observed over the study period was within the expected diurnal fluctuation range. The results suggest that absolute leptin levels were not abnormal, and therefore the relationship with symptom severity might only be observed with a longitudinal design.

While we believe the daily immune monitoring approach is a strong complement to cross-sectional studies, there are still limitations with our small sample size. We note that FM and CFS are frequently co-morbid. Future studies of larger populations would allow us to determine the degree of overlap between these conditions. A larger study would also allow us to study lagged effects and examine possible pathways of causality. Our network analyses, using a simple massive univariate analysis approach, revealed that leptin was the only cytokine to covary significantly with fatigue in participants with CFS, while there was no relationship between fatigue and leptin (or any other cytokine tested) in the healthy controls. Even though leptin is reported to modulate other cytokines, specifically TNF-alpha, IL-2, IL-6, and IL-12 [28], we did not observe relationships between those cytokines and fatigue variability. Future work should address whether leptin is the causal factor in fatigue severity, perhaps by administration of recombinant methionyl human leptin. Future analyses may also use time-lagged variables and techniques such as Granger Causality Modeling to better determine causal pathways.

## Conclusions

These results add to the literature supporting a role of cytokines in CFS pathophysiology. We have identified a subset of women with CFS who demonstrate a strong correlation between leptin levels and fatigue severity. Future work should explore both the role of leptin, and of daily cytokine fluctuations in general, in driving CFS disease severity. Ultimately, these analysis techniques may reveal biomarkers for improved diagnosis, and yield new targets for improved treatments.

## Competing interests

The authors declare that they have no competing interests.

## Authors’ contributions

EAS was responsible for participant recruitment, data collection, and drafting the manuscript. LC was responsible for literature search and manuscript editing. EAS, KSB, and JY were responsible for data analysis. JM was responsible for the diagnosis of CFS and participant recruitment. HM was responsible for supervising the serum assays. IC was responsible for protocol development and medical supervision. JY was the primary investigator and was responsible for obtaining funding, study design, and study supervision. All authors have read and approved the final report.
